# Climate change is a major stressor causing poor pregnancy outcomes and child development

**DOI:** 10.12688/f1000research.27157.1

**Published:** 2020-10-09

**Authors:** David M. Olson, Gerlinde A.S. Metz

**Affiliations:** 1 Department of Obstetrics and Gynecology, University of Alberta, 220 HMRC, Edmonton, Alberta, T6G 2S2, Canada; 2 Canadian Centre for Behavioural Neuroscience, Department of Neuroscience, University of Lethbridge, Lethbridge, Alberta, T1K 3M4, Canada

**Keywords:** Climate change, mental health, mothers, fathers, pregnancy, adverse outcomes, children, developmental trajectories

## Abstract

The climate crisis is the existential threat of our times and for generations to come. This is no longer a threat but a reality affecting us, our children, and the generations that follow. Pregnant mothers, their fetuses, and their children are among those at greatest risk in every population and every jurisdiction. A timely consideration is the health of racialized groups who are particularly vulnerable owing to the confluence of several risk factors that are compounded by climate change. Included among these are Indigenous communities that are the most directly threatened by climate change. This review discusses the main health challenges faced by mothers, fathers, and their children during the climate crisis, focusing on mental health as a causal factor. Exploration of this topic includes the role of prenatal maternal and paternal stresses, allostatic load, and the effect of degradation of the environment and ecosystems on individuals. These will be examined in relation to adverse pregnancy outcomes and altered developmental trajectories of children. The climate crisis is a health threat multiplier that amplifies the health inequities of the most at-risk populations and individuals. It accelerates the increase in allostatic load of those at risk. The path of tragedy begins with an accumulating allostatic load that overwhelms both individual and socio-ecological resilience. This can lead to worse mental health including depression and anxiety and, in the case of pregnant women and their children, more adverse pregnancy outcomes and impaired developmental trajectories for their newborn children. We argue that there is an urgent need to develop new (or re-discover or re-purpose existing) tools that will predict communities and individuals who are experiencing the highest levels of climate-related hazards and intervene to reduce stress and increase resilience in pre-conceptual women and men, pregnant and post-partum women, and their young children.

## Introduction

The earth is warming because of the effects of greenhouse gas emissions, and this warming trend is accelerating and will continue for many years until better controls are put into place, targets are met, and alternative energy sources are commonplace. Even if all nations were to meet the commitments of the 2015 Paris Accord, the global temperature will continue to rise by 1.5 °C by 2100 ^1^. Irrefutable evidence exists that for some time to come there will be an increased number of catastrophic consequences due to the existing climate crisis. There will be more extreme weather conditions (e.g. hurricanes and typhoons), increased air and land pollution, increased ultraviolet radiation, ocean acidification, and rising sea levels. These will lead to more heatwaves, drought, and wildfires, reduced crop yields, food insecurities (reduced agricultural capacity, livestock, and aquaculture productivity), and floods, which along seacoasts will lead to more migration from cities under water. The consequential loss of biodiversity and ecosystem collapse will cause an increased number of pests and vector-borne diseases and a host of adverse health outcomes. We will experience increased risk of non-communicable diseases (NCDs) including obesity, type II diabetes, and heart/cardiovascular issues, more respiratory disease including asthma and pollen allergenicity burden, and more harmful algal blooms. Already it is evident that NCDs increase an individual’s risk for communicable disease (e.g. COVID-19) and vice versa. These problems will be exacerbated by the concomitant climate crisis calamities of loss of human habitation, poverty, mass migrations, and violent conflicts. The richest nations are contributing the most to the crisis, but the poorest nations are bearing the brunt of the consequences. These environmental, social, and health risks will consolidate on worsening mental health and increased morbidity and mortality of humankind and consequent loss of capacity to deal with the crisis ^1, 2^.

The climate crisis is the existential threat of our times and for generations to come. These are no longer threats but realities affecting us, our children, and the generations that follow. At the apex of the health impact pyramid are pregnant mothers, their fetuses, and their children because they are among the most at-risk individuals in every population and every jurisdiction ^3– 5^. Importantly, the health of Indigenous mothers and their children is a particularly critical issue since Indigenous Peoples’ communities are the most directly threatened by climate change ^5, 6^. This review will explore the topic of prenatal maternal stress (PNMS) and paternal stress, allostatic load, and the effect of degradation of the environment on individuals. These will be examined in relation to adverse pregnancy outcomes and altered developmental trajectories of children. This exploration will reveal a public health paradox whereby many of the global gains made in recent decades in maternal–child health will be nullified by the losses due to climate change ^7^.

## Pregnant women and their developing children are one of the groups most at risk of the effects of the climate crisis

The application of intersectionality theory to assess the relative risk of various populations to health inequities due to climate change is an emerging practice ^8, 9^. Diverse subgroups emerge as having greater risk, which include pregnant mothers and their young children along with the elderly ^7^. Critically, though, newborns who are victims of climate change, either directly or indirectly by being the conceptuses/fetuses of women and men affected by climate change, may have an entire lifetime of chronic disease or lost capacity ahead of them. Increasing temperatures and heat exposure and the associated physical stresses increase delivery risk for pregnant women, especially shorter gestations ^10^. In a systematic review where “environmental temperature” and “climate change” were associated with various adverse pregnancy outcomes, all studies that fit the study criteria reported a significant relationship between climate change-related exposures and several adverse pregnancy outcomes: eclampsia, preeclampsia, cataract, low birth weight, preterm birth, hypertension, sex ratio, and length ^11^. The estimation is that 25,000 infants per year between 1969 and 1988 were born earlier than normal (39–40 weeks’ gestation) as a result of heat exposure, with a total loss of 150,000 gestational days per year ^10^. Without intervention, the estimate is that by the end of the century there will be an additional loss of 250,000 days of gestation per year ^10^. The World Health Organization estimates that 88% of the burden of disease attributable to climate change occurs in children younger than 5 years ^12^. Climate change puts children at risk of post-traumatic stress disorder (PTSD), depression, anxiety, phobias, sleep disorders, attachment disorders, and substance abuse, which in turn make them vulnerable to problems with emotional regulation, cognition, learning, behavior, language development, and academic performance ^12^. In addition, these early life experiences have a long-lasting impact on the developmental trajectories also known as “life-course effects” ^13, 14^. For example, when exposed to heat during the prenatal and early life period, adolescents show lower academic attainment and fewer years of schooling ^15^. Moreover, children exhibit high levels of concern over climate change, and these effects and concerns also lead to adverse adult mental health outcomes ^16^. The longer-term effect of PNMS and early childhood stress is the precocious development of NCDs. They account for 71% of deaths in the world and 75% of premature deaths in low- and middle-income countries (LMICs) ^17^.

## Effects of the climate crisis on mental health

In the 2016 Lancet report on sustainable development and global mental health, mental health is identified as the “most neglected of all human health conditions” and a “failure of humanity” ^3, 5, 18^. Berry in 2010 pointed out that climate change may directly affect mental health through exposure of its victims to trauma and indirectly through primary effects on physical health and community wellbeing ^19^. An excellent and exhaustive study of the relationship of current mental health knowledge and studies related to climate change and how healthcare systems can respond has been compiled by Hayes and her colleagues ^5, 20– 22^. In these reports, they emphasize that the amount of work relating climate change to mental health should be strengthened; for instance, at the community level, it remains understudied. In addition, it is just as important to understand the accumulation of stressful events prior to a natural disaster or other climate change-induced environmental event as it is to assess and mitigate the effects of the disaster after the event. On the contrary, not only may the effects of the event result in worse mental illness in some people but also, in different individuals, the event could promote the positive feelings of compassion and solidarity with others who experienced the same tragedy. A better understanding of these relationships is urgently required in order to reduce stress and promote individual and community resilience.

In spite of the work that has been done, the impact of climate change on youth mental health remains relatively unexplored ^23^. Its effects on exacerbating poverty, malnutrition, and disease could serve as independent risk factors for the development of depression among youth, especially those living in developing nations where these are already serious problems. If youth are predisposed to depression and anxiety, then adding the mental health risks due to climate change to this existing burden could synergistically lead to dire outcomes. A unique challenge for youth is that their abilities to cope with these stresses and their levels of resilience are not yet fully developed, meaning that they have less of a buffer for mitigating the mental health risks of a climate disaster. As a consequence, they are more susceptible to climate change trauma and more likely to become depressed than older family members ^23– 26^. Left alone, these stresses may add to the overall allostatic load of youth maturing into adults and persisting into their pre-conception period of life.

A number of new concepts have emerged to better understand, study, and address the mental health impacts caused by climate and environmental change. Solastalgia, coined by Glenn Albrecht ^27^ (a conjunction of “solace” and “nostalgia”), is one of an emerging set of concepts collectively used for describing the effects of climatic and environmental change on mental health and wellness. This set of related emotional concepts has been described as “psychoterratic” or earth-related states ^28, 29^. Solastalgia is the distress caused by the transformation and degradation of one’s home environment, including environmental degradation caused by climate change and associated natural disasters ^30^. It can be applied equally to the mental health effects of environmental erosion of the ice pack on Inuit or to drought on western Australian farmers ^31^. In just a few years, the concept has led to several academic reports and is already a field of investigation.

## Prenatal maternal stress leads to adverse pregnancy, newborn, and generational outcomes

### Prenatal maternal stress and non-communicable diseases

The developmental origins of health and disease (DOHaD) approach ^13^ considers that multiple environmental factors operating on the mother before, during, and after pregnancy and breastfeeding can influence the development of the child in ways that may favor survival in the short term but may also compromise health in the longer term ^14^. Increasing attention has been paid to the environment and experiences of the mother during pregnancy; consequently, PNMS has become an important subject of research. Reviews of the animal ^32– 35^ and human ^36– 38^ research on PNMS suggest that stressing the pre-conception or pregnant person or animal, or maternal exposure to depression or adverse life events in human pregnancy, including adverse childhood experience or chronic abuse ^39, 40^, is associated with a host of negative pregnancy outcomes (e.g. preterm birth, preeclampsia, intrauterine growth retardation, or gestational diabetes mellitus) and adverse developmental trajectories for the newborn. These include especially neurodevelopmental and metabolic disorders such as childhood and adult NCDs ^41– 44^, neurodevelopmental impairments ^45^ including anxiety disorder, conduct disorder, and attention-deficient hyperactive disorder, and even impaired adaptive immunity ^46^. These have disastrous consequences for life-long health ^13, 14^.

Early life exposures impact health and disease risk in a sex-specific manner. Girls face greater psychosocial risks than boys ^47^. While obesity risk is often greater in boys during childhood, women, later in life, are at a greater risk. Unhealthy behaviors and exposure to harmful environments in fathers affects generational transmission by sperm ^48^ and translates into increased NCD risk in offspring. These sex effects are nuanced by sociodemographic factors ^49^ and often contribute to a disproportionately higher burden of NCDs upon women prior to, during, and after pregnancy. Sex often impacts the effectiveness of intervention ^50^.

### PNMS and generational effects

Collectively, the effects of stress appear to be particularly significant in preterm birth risk ^51^. Stress across and among generations can influence allostatic load as a prognostic factor. Factors affecting preterm birth risk can be inherited by the offspring from the maternal line ^52^. Through permanently changing the inflammatory cytokine milieu, prenatal stress in the offspring can lead to pregnancy complications in later life ^53^, be passed on to subsequent generations ^54^, and influence adult disease ^55, 56^ and behavior ^57– 59^. Thus, stress in the mother during pregnancy has the potential to shape physiological responses and adverse birth outcomes over multiple generations.

Studies using pregnant Long-Evans rats examined whether it was repetitive stress in each generation or a single stress to a previous generation that influenced pregnancy and developmental outcomes. They demonstrated that preterm birth risk and metabolic, endocrine, and behavioral consequences were “programmed” by inflicting gestational stress in the F0 parental generation (transgenerational stress) or by stressing each generation (multigenerational stress) ^60^. Stressing every gestating generation led to numerous adverse pregnancy and newborn outcomes that accumulated with every subsequent generation. Interestingly, similar results were observed in the transgenerational model ^60^. The implication of these observations is that PNMS caused by a climate change event can be passed on to succeeding generations through epigenetic means, even to the F3 generation. Therefore, the stress effects of a climate change event will likely last for several generations.

### PNMS and accumulation of stressors: allostatic load

PNMS is characterized by a variety of stresses a pregnant mother can experience and that can impact her offspring’s development and lifelong health. It is associated with a higher risk for neuropsychiatric diseases, such as anxiety and depression, and neurodevelopmental disorders as well as schizophrenia ^61– 63^. It is now widely acknowledged that maternal stress is both intergenerationally and transgenerationally passed on, probably via epigenetic mechanisms ^60, 64– 69^. This transmission is believed to be independent of the stressor’s timing, whether it happens before conception, such as abuse experienced by the mother as a child, or during adult life in the form of interpersonal violence ^65, 70– 72^. PNMS is documented to influence birth and the timing of delivery in addition to increasing the risk of adverse pregnancy outcomes ^39, 73– 75^. Likewise, pre-conceptional stress in the father can epigenetically alter the sperm characteristics to increase the risk of NCDs in generations of offspring ^76^. Considerable data have convincingly demonstrated that natural disasters (e.g. ice storms that disrupt electrical power for weeks or floods) have a profound effect on PNMS, which in turn has considerably affected pregnancy outcomes and child development ^32, 45, 77– 79^.

Stress is inherent to life, pregnancy, and transmission to offspring. This pertains to fathers as well as mothers; the developmental timepoint and type of paternal stress influence the transfer of these phenotypes to offspring and subsequent generations ^80^. Hence, a single stressor is hardly representative of day-to-day life for many pre-conception or pregnant women and their partners. Rather, the most at-risk populations and individuals experience a multiplicity of stressors which collectively add to an individual’s overall stress load, or what is known as allostatic load. These repeated or chronic stresses lead to an increased allostatic load that represents the “wear and tear” of these stressors on the mind and body. When this becomes overwhelming, it can lead to an adverse health outcome ^81, 82^. Clearly, the accumulated stressors due to climate change can lead to increased allostatic load.

Stress has also been shown to increase the susceptibility of an individual to infections and to be associated with a pro-inflammatory profile ^83– 86^. Recently, a “two-hit” model of psychological and inflammatory stress dramatically increased the variability of timing of gestation length in pregnant rats ^87, 88^. It also demonstrated that maternal psychological stress induced anxiety in male and female offspring adults, whereas the inflammatory stress increased exploratory and risk-taking behavior only in adult female offspring. This is another way that climate change can affect fetal/newborn development in that it can alter the environment the fetus is programmed to expect upon birth, creating a metabolic mismatch that alters its newborn and/or adult health.

## Conclusion: addressing the effects of the climate crisis on maternal–child health

Studies have been discussed that suggest that climate change stressors impact fathers as well as mothers, that climate change stressors accumulate, reaching a “tipping point” that can propel the individual away from health and towards adverse pregnancy outcomes and/or adverse offspring development, that climate change stress may be passed on to succeeding generations, and that climate change may lead to a mismatch between the type of environment the fetus was programmed to expect and the type of environment it actually encounters, thereby resulting in altered development and/or adult health.

The climate crisis is a health threat multiplier that amplifies the vulnerability of the most at-risk populations ^5, 89^. It accelerates the increase in allostatic load of the most at risk. The path of tragedy begins with an accumulating allostatic load that overwhelms resilience. This leads to worse mental health including depression and anxiety and, in the case of pregnant women, their partners, and their children, more adverse pregnancy outcomes and impaired developmental trajectories. As confirmation, study after study identifies pregnant women and young children as being one of the most at-risk groups affected by the climate crisis ^3, 4, 89^.

Individuals are generally protected against these adverse health outcomes by one’s personal and/or community resilience. Resilience is defined in both the individual and the socio-ecological context ^90^, which is “the capacity of a socio-ecological system to cope with a hazardous event or disturbance, responding or reorganizing in ways that maintain its essential function, identity and structure, while also maintaining the capacity for adaptation, learning and transformation” ^91^. But the adverse pregnancy and development problems occur when climate change stressors increase allostatic load to a level where one’s resilience, or the resilience of a community, is overwhelmed.

An Australian conceptual framework ^92^ argues that a binary approach is needed to address these climate change effects. At one level, there is a vital need to mitigate the very significant, global, and highly political issues of reducing greenhouse gas emissions, enhancing carbon sinks, switching from carbon fuels to renewable energy sources, reducing pollution, and switching to alternative food sources. At the same time, interventions that more directly address the stress and mental health problems that concern pre-conceptual women and men (high school and university students), pregnant and post-partum women, and their young children require attention. The World Health Organization recommends population-based interventions over those aimed at high-risk individuals, primary over secondary interventions, and controlling distal rather than proximal climate change risks to health ^93^.

Finally, there is a need to create new tools that will predict individuals and communities at the greatest risk and new interventions to reduce these risks, reduce stress, and increase resilience. It is also possible to rediscover “old tools” that work well for some communities who learn how to apply them with the goal of enhancing both individual- and community-level resilience. An example of rediscovering old tools is highlighted by a recent study of Inuit youth aged 15–25 from the Canadian Arctic who are experiencing rapid changes in climate conditions. They identified five factors that are a throwback to more traditional ways used by their community to reduce depression and anxiety and improve mental health and wellbeing. They are 1) being on the land, 2) connecting to Inuit culture, 3) maintaining strong communities, 4) maintaining positive relationships with family and friends, and 5) staying busy ^94^. Learning from community studies like these will open new avenues for better intervention and resilience building for larger population-based strategies and policies that support health in mothers, their children, and future generations.
